# htsint: a Python library for sequencing pipelines that combines data through gene set generation

**DOI:** 10.1186/s12859-015-0729-3

**Published:** 2015-09-24

**Authors:** Adam J. Richards, Anthony Herrel, Camille Bonneaud

**Affiliations:** 1 Station d’Ecologie Expérimentale du CNRS, USR 2936, Route du CNRS, Moulis, 09200 France; 2 UMR 7179 CNRS/MNHN, Département d’Ecologie et de Gestion de la Biodiversité 57 rue Cuvier, Case postale 55, Paris, 75231 France; 3 Ghent University, Evolutionary Morphology of Vertebrates, K.L. Ledeganckstraat 35, Ghent, B-9000 Belgium; 4 Centre for Ecology & Conservation, College of Life and Environmental Sciences, University of Exeter, Penryn TR10 9FE, Cornwall, UK

**Keywords:** Gene set analysis, Gene ontology, RNA-Seq

## Abstract

**Background:**

Sequencing technologies provide a wealth of details in terms of genes, expression, splice variants, polymorphisms, and other features. A standard for sequencing analysis pipelines is to put genomic or transcriptomic features into a context of known functional information, but the relationships between ontology terms are often ignored. For RNA-Seq, considering genes and their genetic variants at the group level enables a convenient way to both integrate annotation data and detect small coordinated changes between experimental conditions, a known caveat of gene level analyses.

**Results:**

We introduce the high throughput data integration tool, htsint, as an extension to the commonly used gene set enrichment frameworks. The central aim of htsint is to compile annotation information from one or more taxa in order to calculate functional distances among all genes in a specified gene space. Spectral clustering is then used to partition the genes, thereby generating functional modules. The gene space can range from a targeted list of genes, like a specific pathway, all the way to an ensemble of genomes. Given a collection of gene sets and a count matrix of transcriptomic features (e.g. expression, polymorphisms), the gene sets produced by htsint can be tested for ‘enrichment’ or conditional differences using one of a number of commonly available packages.

**Conclusion:**

The database and bundled tools to generate functional modules were designed with sequencing pipelines in mind, but the toolkit nature of htsint allows it to also be used in other areas of genomics. The software is freely available as a Python library through GitHub at https://github.com/ajrichards/htsint.

## Background

The use of archived knowledge when analyzing sequencing data is both typical and necessary to navigate the often vast quantities of gene-oriented results produced with high-throughput sequencing technologies. These sequencing techniques, when applied to the transcriptome, are referred to as RNA-Sequencing (RNA-Seq) and their use has taken a key role in transcriptomics [1]. The organization of transcriptomes is naturally modular [2] and the integration of heterogeneous data improves our ability to resolve relevant biological processes [3]. Different forms of experimental data capture different aspects of a larger, more complex biological system. Subramanian and colleagues introduced the method of Gene Set Enrichment Analysis (GSEA), which both emulates the modular nature of biological systems and provides a generalizable framework to integrate multiple sources of data into transcriptomic analysis pipelines [3].

GSEA, or more generally referred to as Gene Set Analysis (GSA), is a diverse group of statistical methods that conceptually can be divided into three general approaches. Arguably the most popular approaches are singular enrichment methods, which can be distinguished from the other methods because they are generally based on the hypergeometric distribution, a Chi-square test, a Fisher’s exact test, or a Binomial probability. For discussion see [4]. Generally these methods are used to test a group of differentially expressed genes for ‘enrichment’ with genes that are annotated with a particular term, which yields a *p*-value for each term. Clearly, singular enrichment methods are useful for understanding how genes in a group are related. However, there are two main concerns: (1) one has to devise a summarizing metric and (2) the relationship among terms is not considered. For example, if we have a group of genes that contain a mixture of annotations consisting of neuron differentiation (GO:0030182), neuron remodeling (GO:0016322) and neuron development (GO:0048666) it is possible that none of the individual terms are significantly enriched, yet the group of genes are undoubtedly related to the progression of neurons over time.

The remaining two approaches for GSA require that there are gene sets, relevant to the goals of the experiment, in hand before analyses are undertaken. Often, these gene sets take the form of curated biochemical pathways, like those maintained by KEGG (Kyoto Encyclopedia of Genes and Genomes [5] or they may be based on an ontology, of which the Gene Ontology (GO) [6] is the standard for most organisms. The distinction between the other two approaches is made between methods that compare values among genes in the set against all other genes in the experiment and those that use the values to test for differences between two phenotypes [7]. There is a lack of consensus among methodologies and suggested ‘best practices’ for GSA methods that require gene sets *a priori*. A number of reviews discuss the topic [8–10].

Resources are abundant for carrying out the functional analyses of gene sets and among them the software DAVID [11] is one of the most frequently used. DAVID is a web-based platform that is accessible to a broad range of users and carries out a number of tasks useful for RNA-seq pipelines including the following: namespace mapping, functional annotation, enrichment analyses, and importantly a variety of ways to navigate the results. In the past decade, the use of functional annotations has experienced unprecedented growth, be it statistical, algorithmic or tool specific [12], but a disproportionately small amount of effort has been placed on the critical step of gene set generation, perhaps helping to explain why singular enrichment methods are more frequently used than *a priori* based ones, despite the perceived advantages of GSA.

htsint is a software library that enables the creation of genes sets independently of the method employed by the user to test these sets for significance. Because gene sets are produced using an unsupervised approach and because it is well-known that high-throughput sequencing results are difficult to interpret, visualization tools were included as part of htsint.

## Implementation

Conceptually, the aim of htsint is to use compiled annotation information from one or more genomes in order to calculate distances among all genes in a specified gene space. This implies a need for efficient annotation querying at the level of taxa. To address this along with name-space mapping, and related organizational tasks, a database is included as an integral part of the library. A common task in bioinformatics is to map gene names to protein space or vice versa and this capacity is facilitated through the database. In addition to the database component there are Gene Ontology, blast mapping, and pipeline components. These three components comprise the core of the library and additional tools are present in a support role.

Python is an efficient programming language for bioinformatics because it is object-oriented, flexible, syntactically clean and there is a growing ecosystem of packages [13]. Recently, the package HTSeq was released to provide a Python-centric environment for high-throughput sequencing pipelines [14]. HTSeq has a developed set of tools for managing reads, assemblies, performing quality assurance, and generation of count matrices. htsint is complementary to HTSeq as it aggregates annotation data specifically for hypothesis testing, while HTSeq and other environments, like Bioconductor [15], facilitate the rest of the pipeline. Convenient packages that include SeqGSEA [16] and GSA [17] exist to carry out the significance testing portion of the pipeline.

### Database

**Fig. 1:**
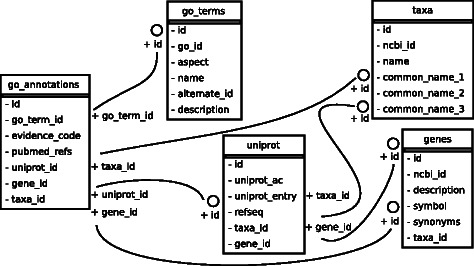
Database entity diagram. Data collected from NCBI, the Gene Ontology, and UniProt are organized for efficient taxa related queries. The database tables or entities are shown along with their attributes. The relationships among tables are designated with edges that connect specific attributes

The database considers by default some of the most commonly studied taxa and for each corresponding gene and protein, GO information is populated either at database initialization or afterwards. The content of the database reflects a list of taxa that are specified in a configuration file. It is possible to populate the database with all available taxa; however, this is not necessary in most cases. The module gathers data, from the National Center for Biotechnology Information (NCBI) ftp://ftp.ncbi.nlm.nih.gov, Uniprot [18] and the GO. The database tables are genes, taxa, uniprot, go_terms, and go_annotations. They are shown as a database schema in Fig. 1. We used the object relational mapper available as part of the Python package SQLAlchemy http://www.sqlalchemy.org to keep the database accessible, extensible, and flexible.

### Gene ontology

The GeneOntology class exists for organizing annotations and building GO graphs. Several helper functions are also available to fetch GO annotations from the database and these are described with examples, in the documentation. The Python package NetworkX [19] is central to the GeneOntology class, because the library is mature enough to handle all graph manipulations natively. GeneOntology is a container for a NetworkX Graph class, which removes much of the burden associated with the graph representation and makes it easier for the general community to develop new algorithms. Furthermore, the visualization of GO networks (or clusters) is simplified through NetworkX as it interfaces with Matplotlib [20], the standard Python library for scientific plotting, and it can export to the powerful network visualization tool Cytoscape [21].

### BLAST

The clustering of genes, based on distances estimated from annotation data, does not directly require homology mapping, but in the context of RNA-Seq pipelines, sequence alignment becomes a necessity. The command line tool, BLAST+, [22] is used either directly or through BioPython [23] to produce a mapping of transcripts against a relevant database like the SwissProt portion of UniProt [18]. The htsint class BlastMapper interfaces these results with the database and pipeline components of the library. The class can be used broadly to summarize the taxa that are associated with an assembled transcriptome or to map between the transcripts and multiple taxa. If multiple taxa are used to create a collection of gene sets, then identifying orthologous and paralogous genes is a required step before GSEA methods may be employed.

### Pipeline

The pipeline to create gene sets is the same for DNA-Seq and RNA-Seq. This process has been generalized so gene space can refer to a genome, a transcriptome or any arbitrary gene space defined by the user. The user first defines a list of genes *x*, as well as a list of taxa *t* to use for functional inference. There are several core classes in htsint that are used to carry out the basic steps involved in the pipeline described below, with a more detailed explanation following.

1. Define the gene space *x*
2. Define the taxa space *t* as the unique taxa from *x*
3. Create a GO scaffold *G* (Fig. 2a)

**Fig. 2:**
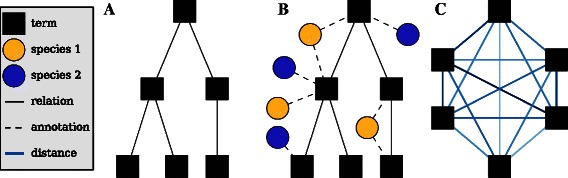
Calculating distances. **a** Represent a specific aspect of the GO (e.g. biological process) as a directed acyclic graph with solid edges corresponding to the *is_a* and *part_of* relationships and nodes representing specific ontology terms. **b** Genes for one or more taxa are added to the network via annotations (dashed edged) and they are used to calculate term-term distances. **c** The term relationships can be re-drawn as a fully connected graph where each weighted edge corresponds to a pairwise shortest path from (**b**). The graph is then represented in gene space as a distance matrix for subsequent clustering
4. Annotate all terms in *G* with terms from *t* (Fig. 2b)
5. Weight the edges of *G* using a measure of semantic similarity
6. Find all pairwise shortest paths in *G* to create *G*_*c*_ (Fig. 2c)
7. Map term-term shortest paths in *G*_*c*_ to gene space
8. Use spectral clustering to partition the genes into clusters

In RNA-Seq pipelines, the gene sets are generally used for significance testing and it follows that genes must be in *x* or have reasonable sequence homology with genes in *x* to be considered for testing. It is useful to BLAST all the genes to be used for testing against *t* so that there is a mapping between the produced gene sets and the genes that will be used for testing. The core of the pipeline is shown graphically in Fig. 2 to highlight how data from multiple species are integrated through the production of gene sets. The entire pipeline, including steps for BLAST mapping, is demonstrated in the tutorial section of the documentation.

As shown in Fig. 2, a GO graph is first created for all the terms in a given GO aspect (e.g. biological_process). Then all annotations corresponding to the genes (*x*) and taxa (*t*) are appended to the graph. Next, the GO graph is trimmed and the edges are weighted by estimating all term-term distances—a computationally intensive step. The distance is based on information theory, where differences in the Information Content (IC) of semantic entities are employed as a measure of the semantic distance [24, 25].

$$ IC(t) = -\ln P(t), $$

such that *P*(*t*) is the number of annotation instances of the term divided by total number of annotation instances from the annotation database. We can then define the semantic distance between a parent-child pair of GO terms as

$$ \text{dist}(t_{p},t_{c}) = |IC(t_{p}) - IC(t_{c})|. $$

GO co-mentioning is included as an optional way to augment the GO graph with edges representing shared gene products [26]. Additional measures of semantic distance will be included in later releases of htsint [27]. Given the graph weighted with semantic distances, all pairwise shortest paths between terms are subsequently computed using a parallel version of Dijkstra’s algorithm [28]. For graphs with more than a few thousand ontology terms, it is recommended to carry out the distance calculations in a parallel environment. The term specific distance matrix is then mapped to a gene specific distance matrix to be used as input in an implementation of the spectral clustering algorithm, a graph partitioning method [29]. Gene to gene distances are set to the shortest path among all possible term-term paths that connect two given genes.

The idea of using spectral clustering in this way is based on previous work where it was shown that the method reasonably partitions gene sets into meaningful functional modules even in the presence of unrelated genes [26]. Additional methodological details and discussion are provided therein where several experiments with pathways and molecular interaction data provide rationale. In particular, there is evidence that multiple sources of annotation information can be combined at the level of affinity matrices to improve the quality of functional modules. The *integration* of arbitrary annotation information (chromosomal location, *cis*-regulatory information, phenotype data etc.) using the scaffolding of the GO is the future of htsint. However, it was necessary to first provide a flexible library with expandable database capabilities before the integration aspects of the package were investigated and implemented. The gene sets produced under this scheme may be referred to as functional modules.

## An example and documentation

Historically, an important vertebrate model for embryology and developmental biology has been *Xenopus laevis*, but because the genome is pseudotetraploid many researchers have turned, in recent years, to the related *Xenopus tropicalis* in order to work with its more tractable diploid genome. The genome of *X. tropicalis* has been published [30] and the developmental transcriptome was detailed more recently [31]. These studies are part of a larger effort that has converged in the form of the *Xenopus* genomics resource Xenbase [32].

In the tutorial example of the htsint documentation, we combine all available functional information for *X. tropicalis* and *X. laevis* at the level of biological_process. All animal related procedures and experiments used to produce the expression data in the tutorial were approved by the Comité Cuvier at the Muséum National d’Histoire Naturelle (Paris, France). To minimize the runtime for the documentation example, we exclude annotations that are inferred electronically, also designated as inferred electronic annotations (IEA). Whether or not IEA annotations should be included when generating gene sets is project and taxa dependent. This is because they will increase the amount of genes included in the gene sets, but they will also make use of non-curated annotations, that may or may not be appropriate for a given project. It should be noted, however, that the reliability of these annotations has improved with recent algorithmic advances [33].

**Fig. 3:**
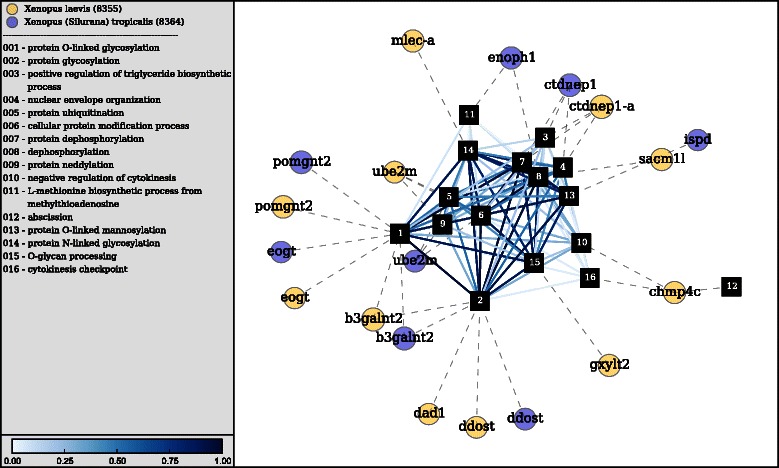
Gene set visualization. htsint was used to visualize a gene set that is produced in the tutorial section of the documentation. Gene Ontology terms are shown as square nodes with the rank according to the number of connections indicated by the label. Additionally, the full name for each term is provided in the legend. Terms are connected by edges representing their semantic distance, which is scaled and shown only for a percentile cutoff (default is 25^th^) for visualization purposes. Genes are represented as circular nodes with NCBI gene symbols overlaid as labels. The gene nodes are connected through annotations and the species from which the gene belongs to is indicated by the color and specified in the legend

The documentation includes an important discussion on parameter estimation and details the entire process that begins with selecting taxa/genes and ends with gene set visualization. The documentation is written using a tool designed for reproducible research, lpEdit [34]. The literate programming style of documentation embeds functional code within the prose of the tutorial. In the tutorial example, a *de novo* assembly of *X. tropicalis* provides context and serves as a working example of htsint. The produced gene sets are tested for significance using GSA [17] and the most significant gene set is visualized. The gene set (shown in Fig. 3) is dominated by glycosylation and other post-translational modifications providing a point of entry for further investigation into genes associated with transcript differential expression.

## Results and discussion

Constructing gene sets from user-specified annotation data is comparable to using the literature to generate hypotheses for testing. For a given organism, partitions provide a functional map of the genome based on accumulated biological knowledge. In general, the gene sets derived from this process provide a faithful description of the annotated (curated or inferred) portion of the transcriptome, but there will remain a percentage of the genes that are not included in significance testing. Although adding well-annotated taxa helps alleviate this problem, this comes at the cost of potentially introducing misleading annotation data. Visualizing gene sets as a network complete with taxa-specific annotation data is a way to evaluate the quality of a gene set based on gene and term functional relationships.

Much of the discussion has been about gene set generation; however, it is important to reiterate that gene set methods themselves offer important advantages over gene-level based analyses. Differential expression can occur as a coordinated change among a group of biologically relevant genes and when these differences are small, then the efficiency of gene-level statistics may be impeded due to a lack of statistical power and a need to correct for multiple comparisons.

A major goal of htsint is to provide users with the ability to explicitly specify taxa (and evidence codes) based on experimental context—that is based on what the domain expert feels is best. We feel that a form of phylogenetic distance is the most biologically meaningful way to select species, but from a practical perspective the annotation coverage will play a more important role in the selection process. Annotation coverage can be calculated using the database and a phylogenetic distance calculator will be soon included as part of the library.

## Conclusion

Now that sequencing technologies are being applied more frequently to non-model organisms, there is a demand for customizable tools that aid functional analyses. Partitioning genes based on information from a list of designated taxa gives the user control over which information is used to generate the gene sets to be used for hypothesis testing. GSA methods are a natural way to test the generated gene sets for statistical significance, and the large number of GSA variants that exist allow generated gene sets to be used in the context of numerous experimental scenarios. Most importantly, this pipeline for gene set creation keeps the analysis intuitive, without requiring detailed algorithmic or mathematical knowledge, as the produced gene sets can be visualized based on connecting annotations and semantic distance. Viewing a pathway, genome or a transcriptome as a set of functionally coherent building blocks is a powerful way to investigate biological phenomena.

## Availability and requirements

**Project name:** htsint**Project home page:** https://github.com/ajrichards/htsint **Operating system(s):** Platform independent **Programming language:** Python **Other requirements:** PostgreSQL, NumPy, NetworkX, SQLAlchemy, Psycopg2, Biopython **License:** MIT **Any restrictions to use by non-academics:** None

## Abbreviations

htsint: High-Throughput data INTegration library
GSA: Gene Set Analysis
RNA-Seq: RNA sequencing
GSEA: Gene Set Enrichment Analysis
GO: Gene Ontology
IEA: Inferred from Electronic Annotation
IC: Information Content
DAVID: Database for Annotation, Visualization and Integrated Discovery
HTSeq: Python package for High-Throughput Sequencing
SeqGSEA: Sequencing Gene Set Enrichment Analysis (R package)
KEGG: Kyoto Encyclopedia of Genes and Genomes
NCBI: National Center for Biotechnology Information
